# Hypertrophic pachymeningitis with cranial nerve palsy as the initial symptom: A case report

**DOI:** 10.1097/MD.0000000000040903

**Published:** 2024-12-06

**Authors:** Xin Zhang

**Affiliations:** a Department of Ophthalmology, The First Affiliated Hospital of Zhejiang Chinese Medical University (Zhejiang Provincial Hospital of Chinese Medicine), Hangzhou, China.

**Keywords:** abducens nerve paralysis, cranial nerve, hypertrophic pachymeningitis

## Abstract

**Rationale:**

Hypertrophic pachymeningitis (HP) is a rare and complex condition marked by inflammation and thickening of the dura mater. It can occur either on its own or as a result of various underlying medical issues. This type of granulomatous meningitis is extremely rare and poorly understood, making diagnosis and treatment particularly challenging. Patients with HP may experience severe headaches and cranial nerve defects, but in some cases, the condition can occur without any symptoms, making it undetected. We detail the case of patient who was diagnosed with HP after presenting with abducens nerve palsy, a condition that significantly affected her vision and quality of life. Remarkably, she showed substantial recovery following a course of methylprednisolone pulse therapy, coupled with careful radiographic diagnosis and follow-up assessments. The decision to report this case stems from its rarity and the diverse clinical manifestations associated with this condition, which can vary widely among patients.

**Patient concerns:**

A 26-year-old Asian female, sought medical attention at The First Affiliated Hospital of Zhejiang Chinese Medical University, where she reported experiencing diplopia, a troubling symptom indicative of abducens nerve palsy.

**Diagnosis:**

To establish a definitive diagnosis, second-generation sequencing biological detection was employed to rule out any infectious causes, while radiographic imaging provided confirmation of HP.

**Interventions:**

In terms of intervention, the patient was promptly initiated on a regimen of methylprednisolone pulse therapy, followed by a carefully monitored oral sequential reduction of the medication. Throughout her treatment, she underwent regular liver function tests to ensure her safety, and liver protective drugs were also administered as a precautionary measure.

**Outcomes:**

As of now, the patient has successfully completed her treatment and is reported to be doing well, marking a positive outcome in her recovery journey.

**Lessons:**

This case underscores the critical importance of imaging examinations in the clinical diagnosis of patients presenting with diplopia, as they play a vital role in both diagnosing and differentiating between various diseases. Furthermore, it is essential to rule out any infection-related factors in patients diagnosed with HP before commencing treatment with glucocorticoids, ensuring a comprehensive approach to patient care.

## 1. Introduction

The most common neurological clinical manifestations include headache, dizziness, or cognitive disorders; notably, all of them are benign, systemic, and nonspecific neurological symptoms. However, the latter could also be early symptoms of more serious neurological syndromes, such as pseudotumor cerebri syndrome or adult idiopathic intracranial hypertension. Both conditions present migraine-like headache, cranial nerve palsy, visual loss or disturbance, pulsatile tinnitus, neck/back pain, and dizziness.^[1]^ In addition, intracranial inflammatory lesions can also cause headache and cranial nerve paralysis. Hypertrophic pachymeningitis (HP) is a rare neurological disorder characterized by localized or diffuse thickening of the cerebral and/or spinal dura mater,^[2,3]^ with the most common manifestations including chronic headache and multiple cranial neuropathies.^[4]^ Although several causative factors have been recognized, including infections (syphilis, tuberculosis) and autoimmune disorders (rheumatoid arthritis, vasculitis syndrome, sarcoidosis, and neoplasms), the etiology of HP remains unclear in some cases.^[5]^ Understanding the underlying causes and clinical manifestations of HP is crucial for improving diagnostic accuracy and therapeutic strategies. HP can present in diverse clinical scenarios, making it a challenge for clinicians to identify and manage effectively. Imaging studies, particularly magnetic resonance imaging (MRI), play a pivotal role in the diagnosis of HP by revealing characteristic thickening of the dura mater and associated changes in the brain structure.^[6]^ However, differentiating HP from other conditions, such as meningiomas, can be challenging and requires careful evaluation.^[7]^ This report presents the case of a young female patient diagnosed with idiopathic HP with symptoms of abducens nerve palsy. Owing to its rarity and clinical manifestations of diversity. Written informed consent was obtained from the patient and the collected data were subsequently used.

## 2. Case presentation

**Figure 1.**
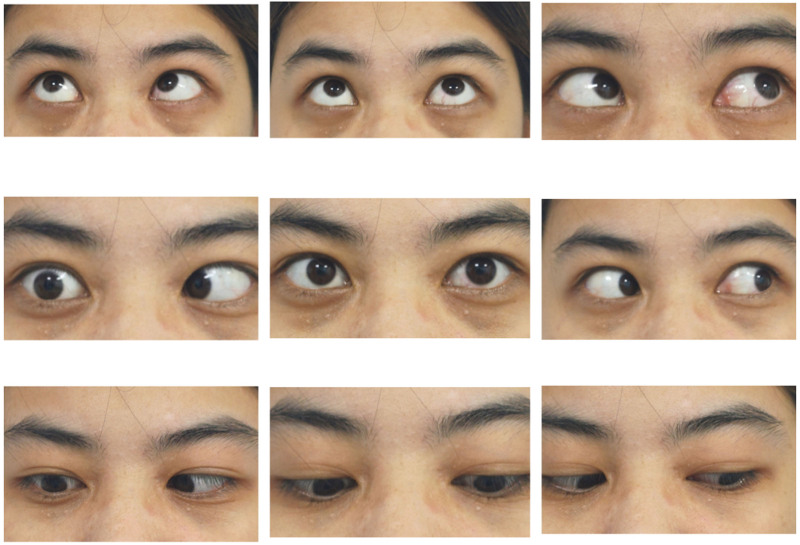
Eye position map in 9 directions. All the abductor muscles were limited to varying degrees. Abduction of the right eye was completely limited.

**Figure 2.**
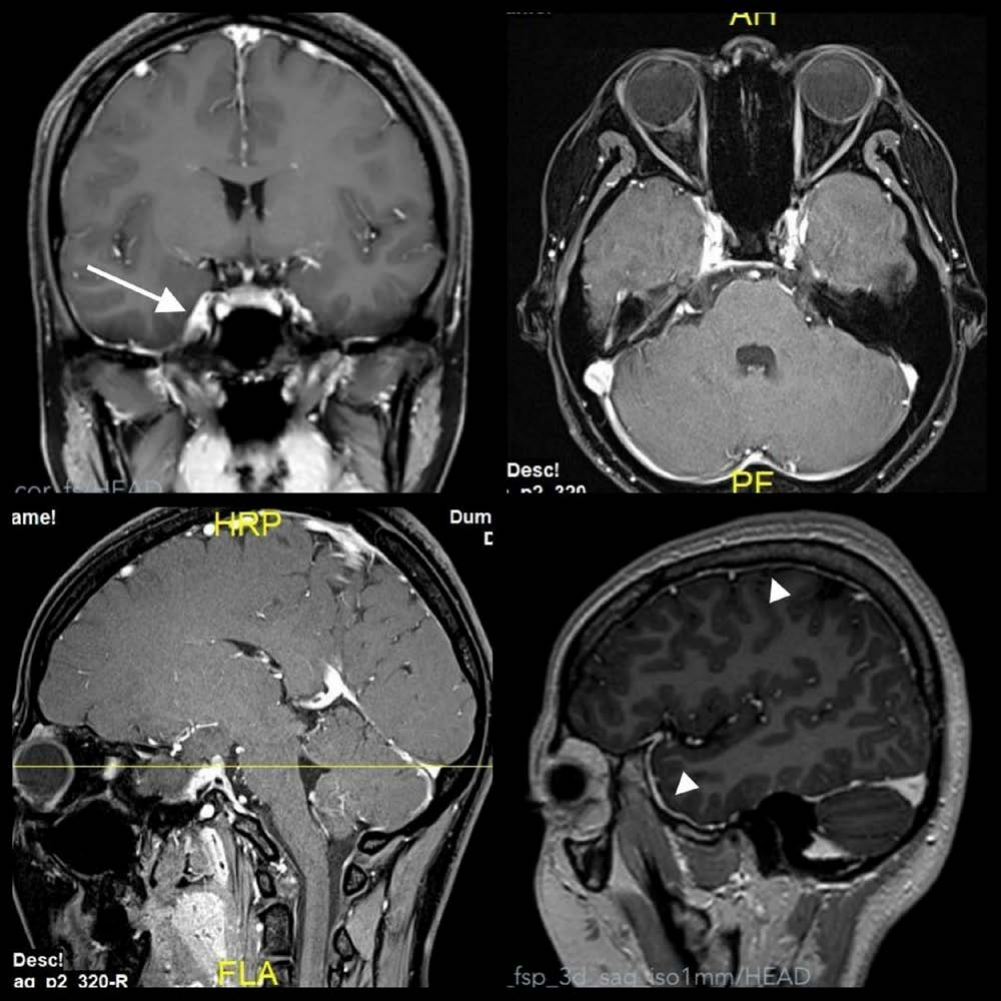
MRI revealed gadolinium enhancement of the thickened prefrontal cortex dura mater (arrowheads) and clivus region near the cavernous sinus on the right side (long arrow). MRI = magnetic resonance imaging.

On July 31, 2023, a previously healthy 26-year-old Asian female who had been suffering from diplopia 11 days. She played in the water and dabbled her lower legs in the stream before the onset of symptoms; however, she did not experience any febrile illness, diarrhea, or vomiting during this period. There was no family history of psychiatric or demyelinating diseases. Her best-corrected visual acuity was 20/20 after wearing the glasses. Examination of the anterior segment and the fundus revealed no abnormalities. Abduction movement of the right eye was limited (Fig. 1). A neurological examination revealed no pathological evidence. In addition to the abnormal liver function tests, no obvious abnormalities were found in routine blood, glucose, antinuclear antibodies, anti-neutrophil cytoplasmic antibodies, etc. Immunoglobulin 4, 25 mg/dL. Examination of the cerebrospinal fluid showed no abnormal indicators. The patient also underwent serum tests for anti-aquaporin-4 immunoglobulin and anti-myelin oligodendrocyte glycoprotein immunoglobuin using a cell-based assay. In addition, various hepatitis indicators were tested, and all the results were negative. MRI revealed gadolinium enhancement of the thickened prefrontal cortex dura mater and the clivus region near the cavernous sinus on the right side (Fig. 2). A single strain of *Chlamydia psittaci* was detected in the serum of the patient by metagenomic DNA analysis of pathogenic microorganisms, but its relative abundance was only 4.77%. Other potential vascular, infiltrative, compressive, toxic, and metabolic causes were also ruled out. We also excluded serious pathologies of the neck can potentially result in cranial nerve palsy.^[8,9]^

The patient’s symptoms significantly improved after intravenous methylprednisolone treatment (500 mg/day for 3 days, followed by 250 mg/day for 3 days). Because the patient experienced drug-induced liver dysfunction following the administration of methylprednisolone, she was concurrently prescribed intravenous Monoammonium glycyrrhizinate cysteine injection and oral liver protectant medications compound glycyrrhizin tablet. Subsequently, the patient was prescribed prednisolone tablets at a dose of 1 mg/kg (10 tablets). These doses were gradually tapered to 10 mg per week. When prednisolone tablets were reduced to 30 mg (6 tablets), the dose was decreased by 5 mg (1 tablet) per week until drug discontinuance. Liver function was monitored regularly during steroid therapy until discontinuation of steroid therapy after 3 months.

**Figure 3.**
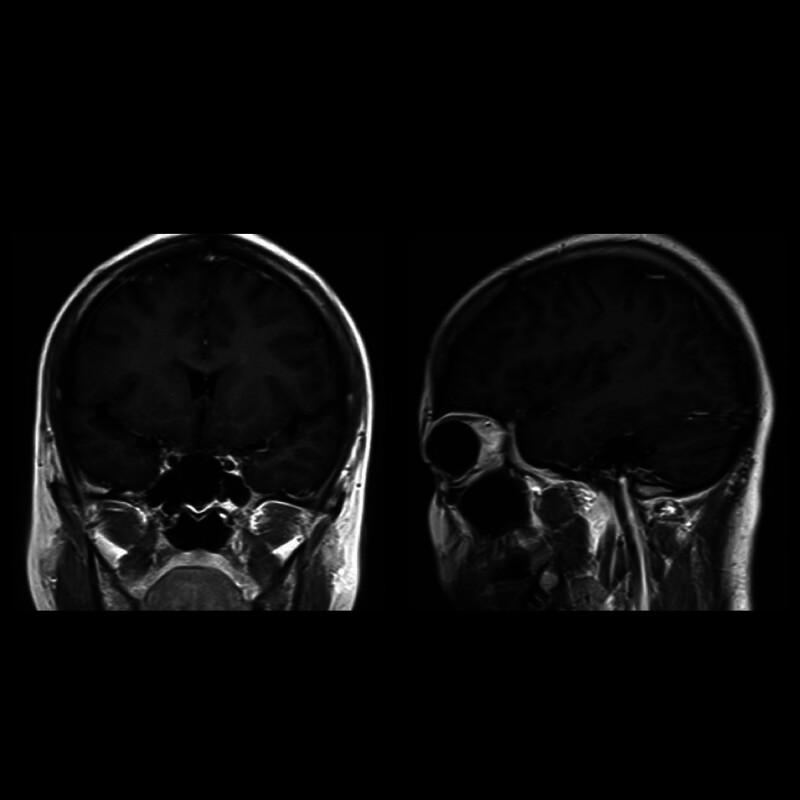
MRI showed fewer signal abnormalities and no new lesions after treatment. MRI = magnetic resonance imaging.

**Figure 4.**
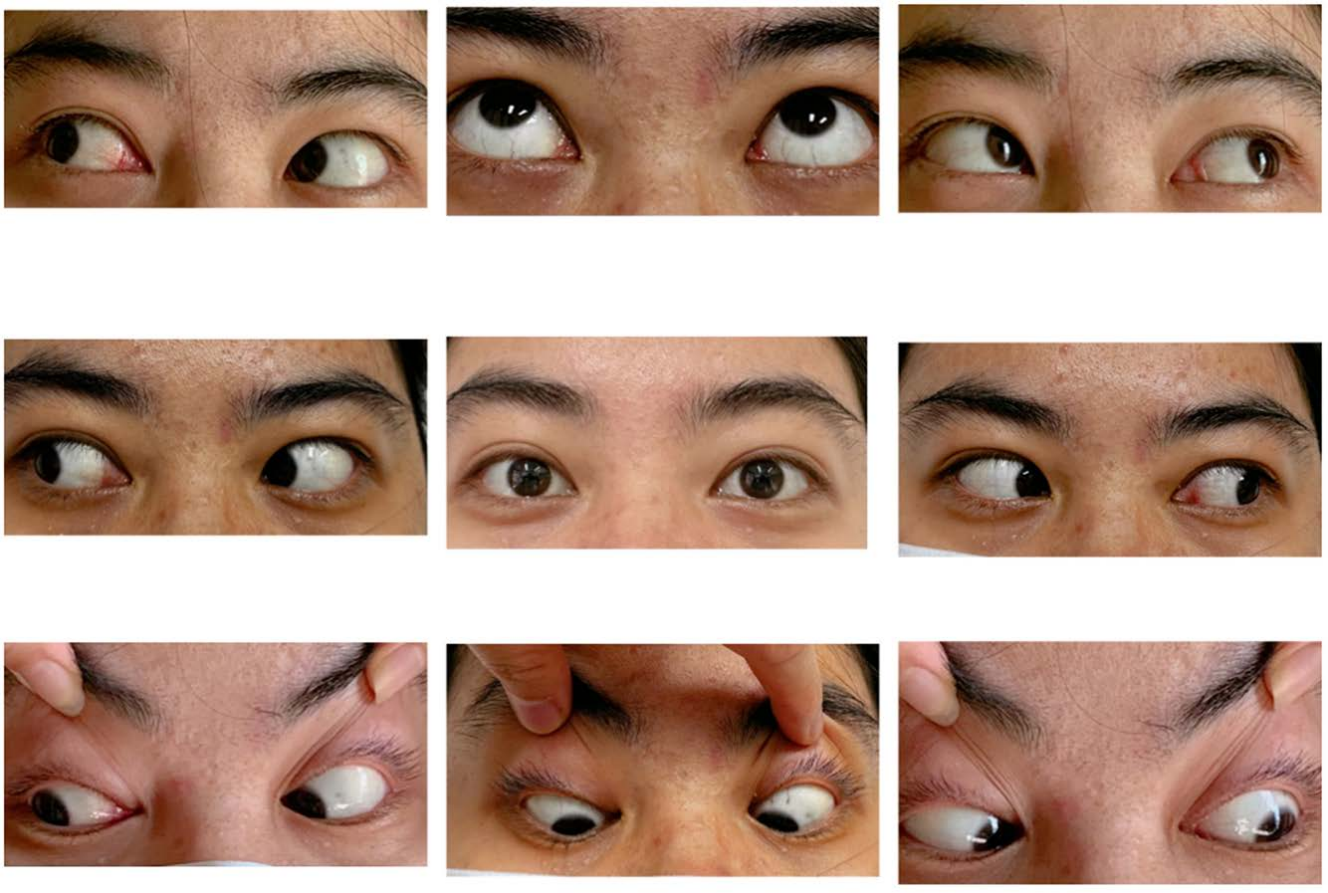
After 3 months of treatment, the 9-position eye map had reached the corrected movement position.

Follow-up at T = 1 month, there was a marked improvement in the abduction of the right eye. However, diplopia was still present when looking at a distance of 5 m. Repeat brain MRI showed fewer signal abnormalities and no new lesions (Fig. 3). The patient was informed that there is a possibility of recurrence of this type of disease and that immunosuppressive agents should be used if necessary. Follow-up at T = 3month, abduction was fully restored, diplopia disappeared (Fig. 4). Follow-up at T = 1 year, her condition was stable. The patient was not taking any immunosuppressive medications.

## 3. Discussion

HP is generally regarded as a rare inflammatory disease that results in the diffuse thickening of the dura mater. HP can be idiopathic or secondary to other diseases. Several causes of secondary HP have been recognized, including infectious diseases, trauma, neoplasms, and autoimmune disorders.^[10–12]^ The diagnosis of idiopathic HP depends on the exclusion of possible etiologies for meningeal thickening. Chronic headache accompanied by multiple unexplained cranial nerve paralysis is the main presentation of HP; however, hypertrophic encephalitis with simple cranial nerve palsy as the first symptom is still rare. The patient had a rapid onset but no headache or neurological symptoms except for abducens nerve palsy in the right eye. The cerebrospinal fluid test also did not suggest any abnormal indicators. Upon inquiry of medical history, the patient had a history of raising birds when she was a teenager (primary school), usually living in hilly areas, and usually eating free-range birds at home. The uncle had a history of predation by birds. Although 1 strain of *Chlamydia psittaci* was detected in metagenomic DNA testing of pathogenic organisms, its relative abundance was very low, and the patient did not have any signs of infection; therefore, an infection association was not considered. The abducens or sixth cranial nerve is purely motor and runs over a long course from the brainstem to the lateral rectus. Through the inferior petrosal sinus, through the Dorello canal, and into the cavernous sinus.^[13]^ Finally, a diagnosis of hypertrophic encephalopathy was suggested based on typical enhanced MRI findings. Imaging in itself cannot be used to discern between the different etiologies of HP; therefore, definitive diagnosis relies on biopsy.^[14]^ However, in this case, definitive diagnosis did not depend on biopsy to avoid the risk of unnecessary craniotomy. According to the imaging features and sufficient clinical manifestations, empirical treatment was given and satisfactory results were obtained.

To the best of our knowledge, there is no consensus regarding the treatment of HP. Previous studies have suggested that corticosteroids are the first choice of treatment for HP. The common method is to start with pulse therapy; then, the dose of corticosteroid is gradually decreased and eventually ceases all together after the symptoms have been relieved.^[15]^ Steroid-sparing agents have been used following disease flares including methotrexate (20 mg/wk), azathioprine (100–200 mg/d), mycophenolate mofetil (1000 mg twice daily), and cyclophosphamide (either oral (100 mg/d) or intravenous (1 g/m^2^/mo).^[16]^

## 4. Conclusion

HP is a reactive inflammatory process affecting the dura mater, which is identified by the thickening and enhancement of the dura mater on contrast-enhanced imaging. In addition to headaches, pure abducens nerve palsy is also a symptom. Steroids and other anti-inflammatory agents are useful for reducing the symptoms of HP.

## Acknowledgments

I would like to thank the patient and her family for supplying the data.

## Author contributions

**Conceptualization:** Xin Zhang.

**Data curation:** Xin Zhang.

**Funding acquisition:** Xin Zhang.

**Writing – original draft:** Xin Zhang.
